# LeafCutterMD: an algorithm for outlier splicing detection in rare diseases

**DOI:** 10.1093/bioinformatics/btaa259

**Published:** 2020-04-21

**Authors:** Garrett Jenkinson, Yang I Li, Shubham Basu, Margot A Cousin, Gavin R Oliver, Eric W Klee

**Affiliations:** Center for Individualized Medicine, Mayo Clinic, Rochester, MN 55902, USA; Department of Health Sciences Research, Mayo Clinic, Rochester, MN 55902, USA; Section of Genetic Medicine, Department of Medicine, Chicago, IL 60637, USA; Department of Human Genetics, University of Chicago, Chicago, IL 60637, USA; Center for Individualized Medicine, Mayo Clinic, Rochester, MN 55902, USA; Department of Health Sciences Research, Mayo Clinic, Rochester, MN 55902, USA; Center for Individualized Medicine, Mayo Clinic, Rochester, MN 55902, USA; Department of Health Sciences Research, Mayo Clinic, Rochester, MN 55902, USA; Center for Individualized Medicine, Mayo Clinic, Rochester, MN 55902, USA; Department of Health Sciences Research, Mayo Clinic, Rochester, MN 55902, USA; Center for Individualized Medicine, Mayo Clinic, Rochester, MN 55902, USA; Department of Health Sciences Research, Mayo Clinic, Rochester, MN 55902, USA

## Abstract

**Motivation:**

Next-generation sequencing is rapidly improving diagnostic rates in rare Mendelian diseases, but even with whole genome or whole exome sequencing, the majority of cases remain unsolved. Increasingly, RNA sequencing is being used to solve many cases that evade diagnosis through sequencing alone. Specifically, the detection of aberrant splicing in many rare disease patients suggests that identifying RNA splicing outliers is particularly useful for determining causal Mendelian disease genes. However, there is as yet a paucity of statistical methodologies to detect splicing outliers.

**Results:**

We developed LeafCutterMD, a new statistical framework that significantly improves the previously published LeafCutter in the context of detecting outlier splicing events. Through simulations and analysis of real patient data, we demonstrate that LeafCutterMD has better power than the state-of-the-art methodology while controlling false-positive rates. When applied to a cohort of disease-affected probands from the Mayo Clinic Center for Individualized Medicine, LeafCutterMD recovered all aberrantly spliced genes that had previously been identified by manual curation efforts.

**Availability and implementation:**

The source code for this method is available under the opensource Apache 2.0 license in the latest release of the LeafCutter software package available online at http://davidaknowles.github.io/leafcutter.

**Supplementary information:**

Supplementary data are available at *Bioinformatics* online.

## 1 Introduction

Next-generation sequencing is revolutionizing the diagnosis and study of rare diseases. Whole exome sequencing has now become standard practice for patients with a suspected rare genetic condition (Posey *et al.*, 2016; Sawyer *et al.*, 2016; Yang *et al.*, 2013). In a landmark paper, Cummings *et al.* (2017) demonstrated the value of RNA-seq for rare disease diagnosis by using multiple RNA-seq analyses to increase diagnostic yield by 35% within a cohort of rare disease probands who received no diagnosis through exome sequencing. Detection of aberrant splicing helped solve a large fraction of these previously unsolved cases. However, the use of *ad hoc* filters and thresholds to detect outlier splicing events in that study is prone to producing large lists of putatively disrupted genes, which requires laborious manual curation and expert knowledge to generate a shortened list of likely causal disease genes. The LeafCutter algorithm (Li *et al.*, 2018) was developed using a Dirichlet-Multinomial generalized linear model (DM-GLM) for differential RNA splicing detection between groups of samples (e.g. to explore splicing differences between tissues sequenced across many individuals). Prior to LeafCutter’s final publication, a second study (Kremer *et al.*, 2017) utilized the algorithm in a one-versus-the-rest fashion to detect outlier splicing in individual probands affected by rare genetic disease. Although LeafCutter was developed with the goal of rigorously modeling variability in counts between groups of samples, it was not designed for the one-versus-many sample comparisons used by Kremer *et al.* (2017) to detect aberrant splicing events in single proband samples.

We found that applying a group comparison method to perform outlier detection is statistically misspecified and thus lacks power compared to a tailored outlier detection test. In brief, statistical comparisons between groups are justified (i.e. consistent) when the sample sizes are kept nearly balanced and the number of observations in both groups grows asymptotically. In the case of a cohort consisting of *N* rare disease samples, the most suitable approach for detecting aberrant splicing events using a group comparison tool is to compare each individual disease sample to the remaining *N* – 1 samples, resulting in the most imbalanced comparison possible; this was the approach pursued in the literature (Kremer *et al.*, 2017). However, even as the cohort grows to an arbitrarily large size, one of the groups will have a single sample, resulting in an estimator that is asymptotically inconsistent. In this article, we present LeafCutter for Mendelian disease (LeafCutterMD), a mathematically rigorous outlier detection procedure to reliably detect aberrant splicing events within a cohort of rare disease probands. The source code will be made available as a new module of the LeafCutter software package. The updated package will enable users to utilize RNA sequencing data to efficiently perform either outlier splicing detection using the proposed LeafCutterMD algorithm, or standard group splicing comparisons (e.g. tissue versus tissue or wild-type versus common variant) using the previously published (Li *et al.*, 2018) LeafCutter methodology.

## 2 Materials and methods

### 2.1 Dirichlet-multinomial model

LeafCutterMD uses the intron-based clustering approach from LeafCutter (Li *et al.*, 2018), wherein splicing is measured as the excision of introns (instead of the inclusion of exons). Biological differences in splicing are thus captured by differing measurements of intron excision. Briefly, split reads anchored by at least 6 nt into each exon are used to specify and quantify excision counts of each intron, which are defined by the regional gap in the split read. LeafCutter then constructs a graph whose nodes are introns connected by edges representing a shared splice junction between two introns. An iterative filtering and graph building approach is followed until convergence, at which point the connected components of the resultant graph define ‘clusters’ of introns (Li *et al.*, 2018). This procedure results in each intron cluster *c* having *I* possible introns indexed i∈I:={1,…,I} in a total of *S* proband samples indexed s∈S:={1,…,S}. For ease of notation, we focus in the subsequent on a single cluster *c* within our *C* total clusters with the understanding that each cluster will be considered independently.

Subsequent to clustering, LeafCutter outputs the counts ${\tilde{n}}_{is}$ for intron *i* and sample *s*, which can be viewed as an *I *×* S* matrix. To regularize the count data, which often has large number of zeros or small values, we apply Laplace smoothing on the data and consider our counts to be $n_{is}:={\tilde{n}}_{is}+1$. We formulate the outlier splicing detection problem as identifying *n_is_* that indicate an abnormally high or low usage of intron *i* in a sample *s* compared to the remaining samples from the cohort. A convenient representation of this problem is to view all intron counts from a sample as a vector $n_{s}={\left(n_{1s},n_{2s},\ldots ,n_{Is}\right)}^{T}$. If all samples were expected to use intron *i* with probability *p_i_*, then the vectors $n_{s}$ would be drawn from a Multinomial distribution $M(N_{s},p)$ where $N_{s}=\sum_{i=1}^{I}n_{is}$ is the total observations (i.e. split RNA-seq reads) in the cluster for sample *s*, and $p={\left(p_{1},p_{2},\ldots ,p_{I}\right)}^{T}$ is the probability of usage for each intron. In general, however, we do not expect all samples to have identical usage probabilities p. Biological variability across individuals will result in variable intron excision rates, and therefore modeling the probability distribution $p^{\left(s\right)}$ for each individual has been shown to work well. A flexible and computationally convenient choice for modeling this distribution over p is the Dirichlet distribution $D(\alpha_{1},\ldots ,\alpha_{I})$ which has *I* parameters that we denote $\alpha :={\left(\alpha_{1},\ldots ,\alpha_{I}\right)}^{T}$.

The leads to the following statistical model for counts vectors $n_{s}$
 

$$\begin{array}{c} n_{s}|N_{s},p∼M(N_{s},p)\\ p|\alpha ∼D(\alpha_{1},\ldots ,\alpha_{I})\; \end{array}$$

whose probability distributions are given by

$$\begin{array}{c} P(n_{s}|N_{s},p)=\frac{N_{s}!}{n_{i1}!\;\cdots \;n_{iS}!}\prod_{i=1}^{I}p_{i}^{n_{is}}\\ P(p|\alpha )=\frac{1}{B(\alpha )}\prod_{i=1}^{I}p_{i}^{\alpha_{i}-1} \end{array}$$

where B(α) is the multivariate beta function that serves as a normalizing constant for the Dirichlet distribution. $P(n_{s}|N_{s},p)$ is a compound distribution that can be combined by integrating out the latent probabilities p as

$$\begin{array}{c} P(n_{s}|N_{s},\alpha )=\int_{p}P(n_{s}|N_{s},p)P(p|\alpha )dp\\ =\int_{p}\frac{dp\cdot N_{s}!}{B(\alpha )\cdot n_{i1}!\;\cdots \;n_{iS}!}\prod_{i=1}^{I}p_{i}^{n_{is}+\alpha_{i}-1}\\ =\frac{N_{s}!\;\Gamma (A)}{\Gamma (A+N_{s})}\prod_{i=1}^{I}\frac{\Gamma (\alpha_{i}+n_{is})}{n_{is}!\;\Gamma (\alpha_{i})} \end{array}$$

where $A=\sum_{i=1}^{I}\alpha_{i}$. The resulting distribution is known as the Dirichlet-Multinomial distribution $DM(N_{s},\alpha )$.

The derivation of the Dirichlet-Multinomial helps us note that a smaller *A* results in a more over-dispersed $DM(N_{s},\alpha )$ compared to the Multinomial with the same mean $M(N_{s},p)$ with $p_{i}=\alpha_{i}/A$. Thus, as A→∞ and $\alpha_{i}/A$ remain fixed, $DM(N_{s},\alpha )\to M(N_{s},p)$. The convergence of the Dirichlet-Multinomial to a Multinomial represents the case where the variability of a sample population around p shrinks to zero, or equivalently the case where the Dirichlet distribution $D(\alpha_{1},\ldots ,\alpha_{I})$ converges to a point mass at p. In the context of outlier splicing events, clusters with very small *A* represent splicing events with a large amount of natural variation in the usage of the various introns, implying that the detection of an outlier would require an especially large deviation from the expected value of the count $E[n_{is}]=N_{s}\alpha_{i}/A$. Conversely, if *A* is large then even a relatively small departure from the expected number of counts $E[n_{is}]$ would be indicative of aberrant splicing.

The other parameter that affects outlier detection is *N_s_*. Small values of *N_s_* indicate small numbers of observations in sample *s*, and the Multinomial portion of the Dirichlet-Multinomial controls the variability due to statistical sampling. Thus, *n_is_* can deviate from $E[n_{is}]$ substantially if the number of observations, *N_s_*, is small, which makes departures from the mean common due to statistical chance. The Dirichlet-Multinomial model therefore accounts for both biological variability in the population, and the uncertainty of the statistical sampling process that produces our observations.

### 2.2 Modeling a one versus all outlier splicing test

A statistical test that determines how unlikely the count *n_is_* comes from the distribution $DM(N_{s},\alpha )$ must estimate the parameters ${\hat{\alpha }}^{\left(s\right)}$ that capture the variability in the proportional intron usage within the population that does not include our sample of interest *s* (s′≠s). Thus, the null hypothesis that underlies the statistical test in LeafCutterMD is that *n_is_* is drawn from the same distribution as the rest of the samples s′≠s, whereas the rejection of the null hypothesis indicates that *n_is_* is an outlier from this population.

To compute the *P*-value of this hypothesis test, we first marginalize the Dirichlet-Multinomial to find the distribution of this particular intron count under the null hypothesis

$$\begin{array}{c} P\left(n_{is}|N,{\hat{\alpha }}^{\left(s\right)}\right)=\sum_{n_{i\prime s},i\prime \neq i}P\left(n_{s}|N_{s},{\hat{\alpha }}^{\left(s\right)}\right)\\ =\left(\begin{array}{c} N_{s}\\ n_{is} \end{array}\right)\frac{B\left(n_{is}+{\hat{\alpha }}_{i}^{\left(s\right)},N_{s}-n_{is}+\sum_{i\prime \neq i}{\hat{\alpha }}_{i{\;}^{\prime }}^{\left(s\right)}\right)}{B\left({\hat{\alpha }}_{i}^{\left(s\right)},\;\sum_{i\prime \neq i}{\hat{\alpha }}_{i{\;}^{\prime }}^{\left(s\right)}\right)}\\ \Rightarrow n_{is}|N_{s},{\hat{\alpha }}^{\left(s\right)}∼BB\left(N_{s},\;{\hat{\alpha }}_{i}^{\left(s\right)},\;\sum_{i\prime \neq i}{\hat{\alpha }}_{i{\;}^{\prime }}^{\left(s\right)}\right) \end{array}$$

where B(·,·) is the beta function and BB(N,α,β) is a Beta-Binomial distribution which is the one-dimensional analogue of—and marginal distribution for—the Dirichlet-Multinomial (Danaher, 1988). The distribution BB(N,α,β) represents the compound distribution where a Binomial trial with *N* samples is drawn with a probability of success *p* that was itself drawn from a Beta distribution with parameters *α* and *β*. We can compute the right *P*-value using the tail probability that a count of *n_is_* or larger is observed from sampling this distribution. Similarly, we can compute the left *P*-value that a count of *n_is_* or smaller is observed from sampling this distribution. The two-sided *P*-value is computed as twice the minimum of these two numbers.

We note that as the number of samples in the cohort increases, the procedure of estimating ${\hat{\alpha }}^{\left(s\right)}$ for each cluster and each sample becomes increasingly computationally burdensome. However, estimates ${\hat{\alpha }}^{\left(s\right)}$ for all *s* should have only small differences because they differ only by a single observation. Therefore, for computational efficiency, we simply estimate $\hat{\alpha }$ using all samples once to approximate ${\hat{\alpha }}^{\left(s\right)}≃\hat{\alpha }$. In practice, this will make the outlier detection test conservative (i.e. larger *P*-values) because the model with $\hat{\alpha }$ includes the variability from the potential outlier. Thus, this technique of setting ${\hat{\alpha }}^{\left(s\right)}≃\hat{\alpha }$ results in a minor loss of sensitivity for gains in computational efficiency, and this becomes increasingly beneficial as *S* increases.

In some cases, a cluster-level summary *P*-value for each sample may be of interest. The above procedure produces *P*-values $\rho_{i},i\in I$ for each intron in the cluster for a given sample. Thus, a practical choice would be to report the minimum *P*-value across the introns within the sample $\rho_{\text{min}}=\min_{i\in I}\rho_{i}$. However, because this summary would lead to smaller $\rho_{\text{min}}$ by chance for clusters with larger numbers of introns *I*, we compute a cluster-level *P*-value by inferring the null distribution of the minimum *P*-value within the cluster. Under this null hypothesis, the *P*-value distribution for each intron is expected to be uniform, and therefore under the assumption that these tests are independent, the minimum *P*-value would be distributed as a beta distribution $\rho_{\text{min}}∼B(1,I)$. Computing the approximate *P*-value for $\rho_{\text{min}}$ from the left tail of the beta distribution can therefore serve as a cluster summary *P*-value when needed.

We use the R bioconductor library Dirichlet-Multinomial (Holmes *et al.*, 2012) to estimate the parameters $\hat{\alpha }$ from the counts $n_{is},i=1,\ldots ,I,s=1,\ldots ,S$, and the R library TailRank (Coombes, 2018) to compute the tail probabilities of the Beta-Binomial distribution. Rankings by *P*-value are computed using the R function rank, which reports ties to have their average rank.

### 2.3 Ethical compliance

The probands and families provided written informed consent to a research protocol approved by the Mayo Clinic Institutional Review Board for this study.

### 2.4 Study subjects

All probands were clinically referred to the Mayo Clinic Center for Individualized Medicine, seeking genetic diagnosis of a suspected rare inherited disease. Probands not fully diagnosed by exome sequencing were selected for whole-transcriptome RNA sequencing.

### 2.5 RNA-sequencing

Sequencing was conducted on blood for 128 individuals. Blood-derived RNA was obtained by collecting peripheral whole blood in PAXgene blood RNA tubes and using the QIAcube system (Qiagen) according to the manufacturer’s protocol for RNA extraction.

Sequencing libraries were prepared with the TruSeq RNA Access Library Prep Kit (Illumina, San Diego, CA). Paired-end 101-basepair reads were sequenced on an Illumina HiSeq 2500 using the TruSeq Rapid SBS sequencing kit version 1 and HCS version 2.0.12.0 data collection software. A median of approximately 200 million reads was generated per individual. Base calling was performed using Illumina’s RTA version 1.17.21.3.

## 3 Results

We compared the performance of LeafCutterMD to the standard LeafCutter likelihood ratio-based approach (Kremer *et al.*, 2017; Li *et al.*, 2018), which represents the current state-of-the-art. We begin with a simulation study where the ground truth is known, and then proceed to examine the performance of both approaches in real examples from a rare disease cohort from the Mayo Clinic Center for Individualized Medicine.

### 3.1 Simulated data

#### 3.1.1 Exon skipping and cryptic exon inclusion

**Fig. 1.**
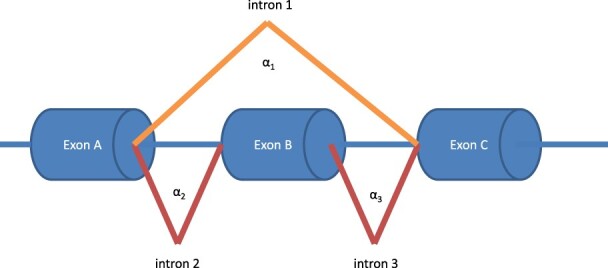
The simulated exon skipping event where the canonical splicing is represented by the two red splicing events, whereas the skipping is represented by the orange splicing event. Note this same setting can represent a cryptic exon when the canonical splicing is given by the orange lines and the proband's splicing is represented by the red lines. The simulation parameters a1, a2 and a3 are shown next to their corresponding introns

We first considered a simple three intron cluster (Fig. 1) that can represent an exon skipping event or inclusion of a cryptic exon. In our first simulation, we assume a low-level background rate of exon skipping in a cohort of 100 samples, with a proband exhibiting dramatically increased rate of exon skipping.

To simulate this possible scenario, the total observations in each sample *N_s_* were drawn from a Poisson distribution with a mean of *N* reads. The reads from a healthy individual were simulated using Dirichlet parameters of $\alpha_{1}=1$ and $\alpha_{2}=N$ and $\alpha_{3}=N$. In this simulation, intron 1 represents exon skipping with Dirichlet parameter *α*_1_, whereas introns 2 and 3 with parameters *α*_2_ and *α*_3_, respectively, represent exon inclusion (Fig. 1). The proband was assumed to have an increased usage of the exon skipping intron, which will be represented by increasing $\alpha_{1}=50$ and drawing samples from a Multinomial with $p_{i}=\alpha_{i}/\sum_{j}\alpha_{j}$.

Of note, this simulation assumes a Dirichlet-Multinomial distribution, which is consistent with both LeafCutterMD and the standard LeafCutter, and thus represents a fair comparison of the methods. We drew 2000 Monte Carlo samples for each scenario with increasing values of *N* representing increasing numbers of supporting reads within the cluster. For each method, we considered the event as being detected if the *P*-value was less than 0.05.

**Fig. 2.**
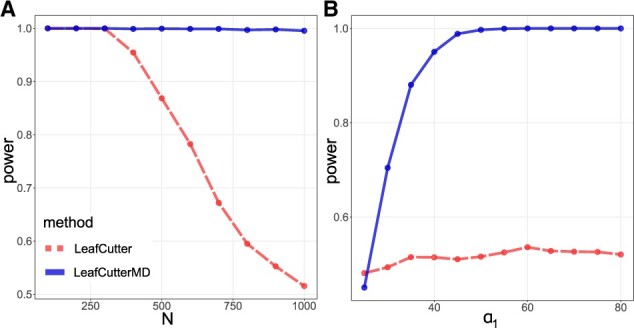
The power in the exon skipping simulation, (**A**) as a function of the average number of reads *N*, and (**B**) as a function of the *α*_1_ effect size, for the proposed LeafCutterMD method versus the state-of-the-art method based on the likelihood ratio test in LeafCutter

Figure 2A illustrates that LeafCutterMD has estimated power (i.e. one minus the probability of a Type II error) of nearly 1 for all values of *N*, whereas, the power of the standard LeafCutter likelihood approach decreases with increasing number of observations. This unintuitive result is due to the fact that reads supporting exon skipping from the single proband sample contribute a smaller and smaller proportion to the likelihood in the learned model, and thus the likelihood ratio test becomes less sensitive to the exon skipping reads, despite their increased presence in the proband compared to healthy individuals. In addition, we found that our proposed LeafCutterMD test is conservative. Indeed, estimated false-positive rates remained below 0.007 for all values of *N* despite a *P*-value threshold of 0.05.

To more comprehensively examine the parameter space of this simulation, we fixed *N *=* *1000 but varied *α*_1_, which for small values indicates a small effect size for the outlier and for larger values indicates a larger effect size. The results in Figure 2B shows that the proposed LeafCutterMD experiences a more rapid gain in power compared to the standard LeafCutter algorithm as effect sizes increase. We note, however, that for very small effect sizes LeafCutterMD’s performance starts to degrade faster than the original method, and this is due to the Laplace smoother, which in practice will reduce the algorithm’s power against very small effect sizes. Such small effect sizes are rarely our concern from a biological perspective when performing outlier detection, and so the reduction in noise and false positives is on the whole beneficial in practice. If increased power against small effect sizes is desirable, the user can remove Laplace smoothing, although reliable recovery of vanishingly small effect sizes at finite sample sizes cannot be expected.

As noted above, the same simulation can alternatively represent cryptic exon detection if we simply set *α*_1_ to be much larger than *α*_2_ and *α*_3_. Thus, consider the same simulation setting as the previous example, but with the population parameters now set to $\alpha_{1}=N,\alpha_{2}=1,\alpha_{3}=1$. The proband will be assumed to have an increased usage of the two cryptic exon introns, which will be represented by increasing $\alpha_{2}=\alpha_{3}=50$ and drawing samples from a multinomial with $p_{i}=\alpha_{i}/\sum_{j}\alpha_{j}$. In this setting, we find that both LeafCutter and LeafCutterMD maintain a power equal to one throughout the simulation parameters.

When considering why there is a discrepancy between the cryptic exon and exon skipping events, we note that the majority of introns (two of three) are affected in the case of the cryptic exon whereas only one of three are affected in the exon skipping event. Because the likelihood ratio test of the original LeafCutter compares the change in likelihood across the entire three intron cluster when allowing all parameters to have unique values in the proband, we expect it to be more sensitive when a larger fraction of the cluster is affected in a given proband. By contrast, when a smaller fraction of introns and reads in a cluster are affected by aberrant splicing in the proband, we expect the original LeafCutter to lose power compared to our proposed LeafCutterMD approach. To test this hypothesis, we include a fourth ‘noise’ intron (i.e. an intron with identical behavior in the proband and the unaffected cohort, that is therefore not relevant to the proband’s phenotype) to this cluster, with increasing read counts such that the event of interest represents a decreasing fraction of the total reads in the cluster. The results are shown in Supplementary Figure S1A and B and demonstrate that in both the cryptic exon and the exon skipping settings, the original LeafCutter experiences a degradation in performance, whereas LeafCutterMD remains robust to the noisy counts. We explore these points further in the next simulation setting.

#### 3.1.2 Growing cluster size simulation

Alternative RNA splicing is a complex and inherently noisy process, producing highly variable recombinations of DNA sequence to form distinct mRNA isoforms. As such, we investigate the role of increasing cluster sizes—which represent increasing isoform complexity—in our ability to the detect outlier splicing. This is critical to the real-world performance of the algorithm as our cohort grows, because clusters can often comprise upward of 15 distinct introns.

**Fig. 3.**
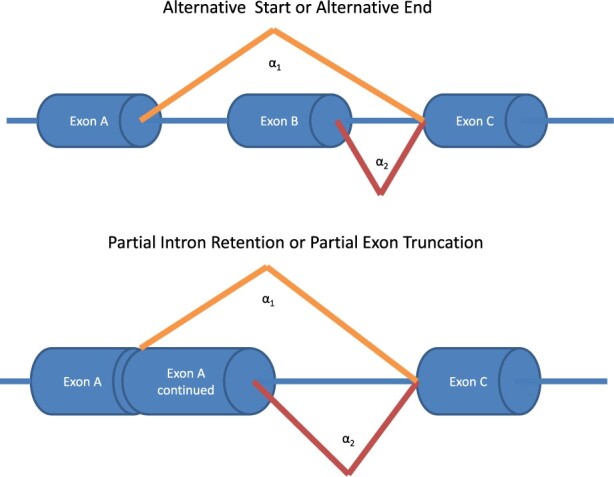
Events that can be represented as a two intron cluster. In our simulation, we include additional ‘noise’ introns to this cluster that have no differential splicing in the proband (unpictured). Increasing the number of reads to this biologically irrelevant portion of the cluster can degrade the performance of the original LeafCutter algorithm, whereas the proposed method is robust to these effects

**Fig. 4.**
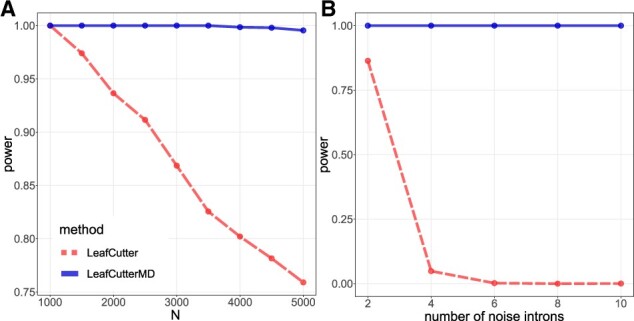
The proposed method retains power even as the number of irrelevant reads *N* is increased, whereas the original LeafCutter method experiences a decrease in power. In (**A**), the reads are all placed in a single noise intron, whereas in (**B**), there is an increasing number of noise introns each receiving 500 additional reads on average

Suppose our event of interest is one of the simple two-intron cases represented in Figure 3, but we will be adding a third intron representing some other unaffected splicing event that is a part of this cluster. We represent this by setting $\alpha_{1}=1$ and $\alpha_{2}=50$ in the cohort and $\alpha_{1}=50$ and $\alpha_{2}=50$ in the proband (indicating a significantly increased usage of intron 1 in the proband), and setting the third noise intron to have $\alpha_{3}=N$ in both the proband and the cohort. The total reads are drawn from a Poisson with a mean of $\sum_{i=1}^{3}\alpha_{i}=N+51$ in both the cohort and the proband, and as before the proband has reads drawn from a multinomial with parameters $p_{i}=\alpha_{i}/\sum_{j}\alpha_{j}$. The results in Figure 4A quantitatively demonstrate the degradation in performance in the original LeafCutter method as compared to the proposed LeafCutterMD methodology, whereas the results in Supplementary Figure S2 explore how this performance is affected by the effect size *α*. Once again, the false-positive rate for the proposed approach never rose above $6.25\times 10^{-4}$ even though the level of the test was set to 0.05, demonstrating the conservativeness of the approach.

To further explore how noisy reads impact performance, we altered the simulation to have an increasing number of noise introns. Each additional intron has *α *= 500 in both the proband and the population, and we increase the number of reads by this amount as well, which results in an average of 500 additional reads going to each additional ‘noise’ intron we add to the cluster. The results in Figure 4B demonstrate that LeafCutterMD is robust against added noise introns, whereas the state-of-the-art method experiences an even more rapid degradation of performance when the noisy reads are spread among more introns in the cluster.

### 3.2 Rare disease cohort

Our simulations demonstrate that the false-positive rate is well controlled. Indeed, the test is conservative as thresholding the *P*-value at a level *α* will results in a false-positive rate strictly less than *α*. Our simulations also demonstrate that LeafCutterMD achieve higher power to detect outlier splicing events compared to the group-comparison statistics in a one-versus-the-rest fashion.

Next, we examine three outlier splicing events from our rare disease cohort that were previously discovered and confirmed by manual review of the cases. We find that LeafCutterMD, but not standard LeafCutter, is capable of detecting these outlier events.

Specifically, we analyzed a cohort of 128 probands with undiagnosed disease following whole exome sequencing (WES). Using RNA-seq data from peripheral blood, three of these 128 cases were identified to have splicing aberrations by manual review of DNA variants and RNA-seq coverage at candidate disease genes prior to any systematic splicing-aware bioinformatic analysis. When we analyzed these RNA-seq data using standard LeafCutter as applied by Kremer *et al.* (2017), we were unable to identify any of the three outlier splicing events. By contrast, we were able to recover all three cases using LeafCutterMD.

#### 3.2.1 Proband 1

**Fig. 5.**
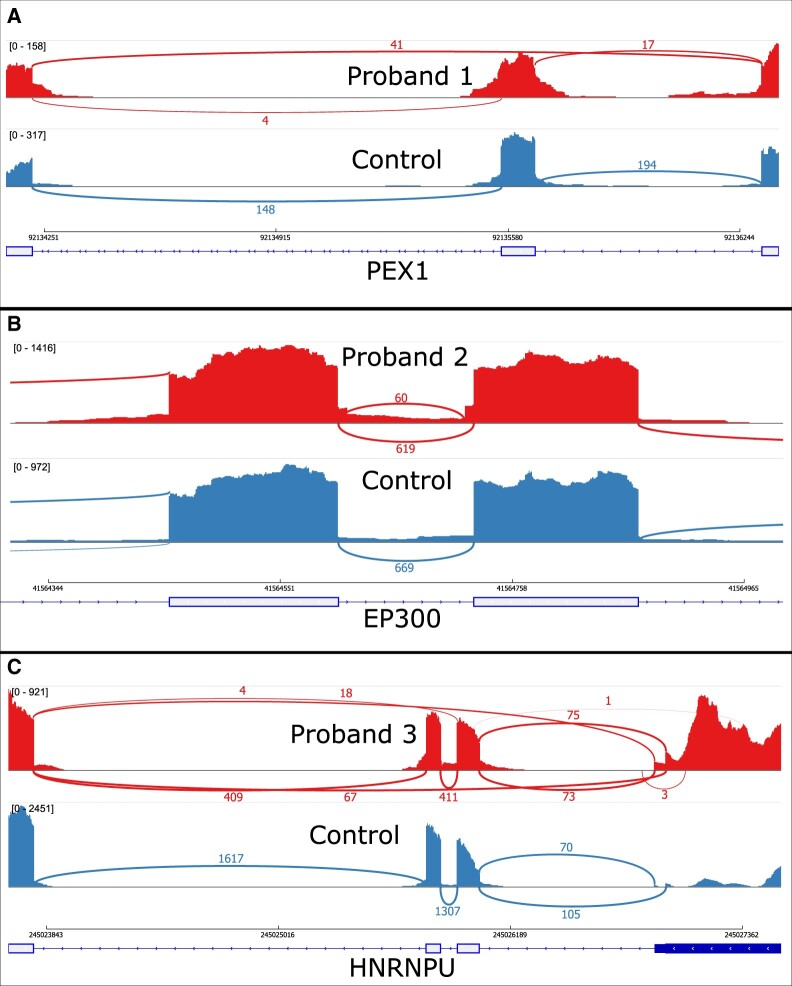
Aberrant splicing in disease cohort. (**A**) Splicing patterns from a representative control sample as well as Proband 1 demonstrate the aberrant pattern of splicing found in this individual in *PEX1*. (**B**) Splicing patterns from a representative control sample as well as Proband 2 demonstrate the aberrant pattern of splicing found in this individual in *EP300*. (**C**) Splicing patterns from a representative control sample as well as Proband 3 demonstrate the aberrant pattern of splicing found in this individual in *HNRNPU*

Proband 1 and her affected sister were born to a consanguineous family and have global developmental delay and refractory epilepsy. WES of the two sisters and their unaffected parents identified a homozygous synonymous SNV (c.1899A>T, p. Arg633Arg) in the penultimate nucleotide of exon 11 in *PEX1* (transcript NM_000466.2) in both girls. SpliceAI (Jaganathan *et al.*, 2019) predicts loss of the exon 11 splice donor (DS_DL = 0.7168, DP_DL=-1) without significant gain of a novel donor (DS_DG = 0.0104, DP_DG=-7). Skipping of this exon was detected by blood whole RNA sequencing, which leads to an out-of-frame transcript that may be a substrate of nonsense-mediated decay. When examined in a sashimi plot (Fig. 5A), a subset of the RNA-seq reads in this proband support abnormal splicing, suggesting a weakening of the splice donor. Further functional work is needed to determine if the c.1899A>T variant is causing disease in this family, however, it is clear that there is aberrant splicing of *PEX1* exon 11, which is not detected by standard LeafCutter as significantly differentially spliced with an adjusted *P*-value of 0.15 although it is ranked as the second most aberrant cluster by *P*-value. In comparison, LeafCutterMD identifies this outlier splicing event with an adjusted *P*-value of $1.3\times 10^{-10}$ and is ranked first by its *P*-value.

#### 3.2.2 Proband 2

Proband 2 has mild global developmental delay, distinctive features, short stature, cerebellar ectopia, Chiari I malformation, hyper-reflexia and attention-deficit hyperactivity disorder with no similarly affected family members. Trio WES identified a *de novo* intronic SNV (c.4026-9A>G) in *EP300* (transcript NM_001429.3). This variant has been seen previously in an unrelated individual with Rubinstein–Taybi (Fergelot *et al.*, 2016), but without any RNA or functional studies. SpliceAI (Jaganathan *et al.*, 2019) predicts moderate loss of the exon 25 splice acceptor (DS_AL = 0.3704, DP_AL = 9) and a strong gain of a novel splice acceptor at c.4026-8 (DS_AG = 0.9788, DP_AG = 1) due to this variant. As visualized in Figure 5B, the abnormal splicing detected by RNA sequencing of blood adds 8 nts to exon 25, causing a frame shift in the transcript. Observation of the variant-induced abnormal splicing provides additional evidence supporting this variant’s pathogenicity, which results in a genetic diagnosis of Rubinstein–Taybi for this patient. Usage of the novel splice acceptor went undetected in a cluster of 8 introns with an adjusted *P*-value of 1.0 using standard LeafCutter. In comparison, the use of the novel acceptor was detected in this cluster using LeafCutterMD with an adjusted *P*-value of $4.1\times 10^{-8}$. Furthermore, the traditional LeafCutter and LeafCutterMD methods, respectively, rank this event by *P*-value in the 28 611th and 154th positions out of a total of 51 347 clusters. Although not a first-place ranking in the proposed method, from the perspective of a rare disease case review, the event is in the top 0.3% of clusters. Indeed, its presence in the first few hundred outlier splicing events is sufficient for manual review, especially when analyzed using common automated or manual phenotypic prioritization and gene annotation techniques in conjunction with genetic variation analysis.

#### 3.2.3 Proband 3

Proband 3 has global developmental delay, focal epilepsy, autism spectrum disorder and downbeat nystagmus with unaffected parents. Trio WES identified a *de novo* 4-bp intronic deletion (c.804-9_804-6delGTCT) in *HNRNPU* (transcript NM_031844.2) predicted to weaken the strength of the exon 3 splice acceptor. *HNRNPU* is associated with autosomal dominant early infantile epileptic encephalopathy 54 and is consistent with the proband’s clinical symptoms. Blood RNA sequencing reveals skipping of both exons 2 and 3 (splicing from exon 1 to 4), as visualized in Figure 5C. This exon skipping event is predicted to lead to an in-frame transcript missing amino acid residues 231–293 and suspected to be disease causal in this individual. The abnormal splice event in *HNRNPU* went undetected in a cluster of 28 introns with an adjusted *P*-value of 1.0 by the traditional LeafCutter method. Comparatively, this was detected by LeafCutterMD with an adjusted *P*-value of 0.03. Similarly, the LeafCutter and LeafCutterMD methods, respectively, rank this event by *P*-value in the 26 539th and 134th positions out of a total of 51 347 clusters. Once again, the new method is able to detect this event from an unbiased case review perspective where it would have been previously undetectable using existing methodologies.

## 4 Discussion

RNA sequencing is becoming an important diagnostic tool for rare disease patients. However, the efficiency and sensitivity of methods for prioritizing disease genes from RNA-seq data have not being systematically assessed. In this article, we present LeafCutterMD, an algorithm for outlier splicing detection in rare disease cohorts. We demonstrate the statistical and practical improvements that result from LeafCutterMD using simulated and real data analyses. The rigorous Dirichlet-Multinomial model that underlies LeafCutterMD accounts for both biological variability in the cohort as well as the uncertainties due to statistical sampling. Our framework naturally accounts for variations in the total number of observations within a cluster, which could vary due to changes in expression levels, or experimental artifacts such as variation in library sizes. Importantly, we have demonstrated by simulation that this updated method outperforms the original LeafCutter when the event of interest is embedded in increasingly large clusters of complex splicing patterns.

We argue that loss of power occurs when applying LeafCutter to a one versus many comparison setting for which it was not specifically designed. Interestingly, as the total read counts grow larger, aberrant splicing events that are supported by a decreasingly smaller fraction of the total number of junction reads are steadily more difficult to detect using the likelihood ratio test implemented in LeafCutter. By contrast, LeafCutterMD maintains high power for all parameters of this simulation. The conservative nature of LeafCutterMD is due to the regularization of the Laplace smoothing as well as the computational approximation discussed in Section 2 whereby the model is fit on all samples including the potential outlier sample. Also as discussed in Section 2, a more powerful, nonconservative test can be used at the cost of added computations by leaving the proband out of the estimation of the parameters from the rest of the cohort. However, the difference between these methods becomes negligible as the cohort size becomes very large. Because the added computational expense scales linearly with cohort size, the less conservative approach is only advised when analyzing a small cohort of probands, where the additional power will be beneficial and the additional computations will be minor.

As the analysis of our patient data indicates, large clusters occur frequently in genes of biological relevance, which can cause the standard LeafCutter to miss events that are identified using LeafCutterMD. We expect this to be an issue of increasing importance as the number of individuals in the cohort increases, because the intron clustering step is performed considering all samples simultaneously. In practice, this can result in increasing cluster sizes as cohorts grow. But these increasing cohorts are important to the outlier detection problem as a large cohort provides a better understanding of normal variability in intron utilization, and thus the robustness to growing cluster sizes is a critical feature of the proposed methodology.

When building a rare disease cohort in which to test for splicing aberrations, there are a few experimental design considerations that will optimize performance. The cohort should be built from individuals with suspected heterogeneous disorders. If many individuals with the same disorder appear in the cohort, it is possible that their aberrant splicing patterns would no longer represent outliers within this cohort; in the case of a group of samples with the same underlying phenotype, the original LeafCutter algorithm should be utilized to compare this group against a group of normal samples. In general, normal samples such as those found in GTEx could be utilized to create or supplement a cohort of samples. But special care should be taken to ensure that different RNA sequencing chemistries do not enter the cohort without some sort of correction in the junction counts to account for the biases between chemistries. For example, a poly-A pull down chemistry might be more biased to observing junctions splicing near the poly-A tail as compared to a targeted capture chemistry. Where feasible, we suggest building a cohort from samples following identical sequencing protocols.

## Supplementary Material

btaa259_Supplementary_Data
