# Supplementary material for: LeafCutterMD: an algorithm for outlier splicing detection in rare diseases
Source: Bioinformatics. 2020 Apr 21;36(17):4609–15. doi: 10.1093/bioinformatics/btaa259 (PMC7750945; doi:10.1093/bioinformatics/btaa259)
Supplement: btaa259_Supplementary_Data [file btaa259_supplementary_data.pdf]

# Supplemental Materials for RareLeafCutter: an algorithm for outlier splicing detection in rare diseases

Garrett Jenkinson [1,2], Yang I. Li [3,4], Shubham Basu [1,2], Margot A. Cousin [1,2], Gavin R. Oliver [1,2,*], and Eric W. Klee [1,2,*]

[1]Center for Individualized Medicine, Mayo Clinic, Rochester, MN, 55902, USA,
[2]Department of Health Sciences Research, Mayo Clinic, Rochester, MN, 55902, USA,
[3]Section of Genetic Medicine, Department of Medicine, University of Chicago, Chicago, IL 60637, USA,
[4]Department of Human Genetics, University of Chicago, Chicago, IL 60637, USA.
*To whom correspondence should be addressed.

## 1 Supplemental Figures

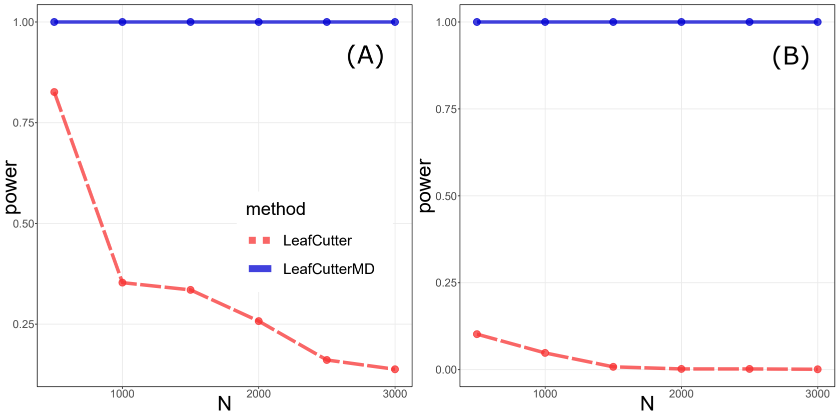


Figure S1: Power results after adding a fourth "noise" intron with $\alpha_4 = N$ average reads of support to (A) the Figure 1 cryptic intron simulation with cohort values of $\alpha_1 = 500, \alpha_2 = 1, \alpha_3 = 1$, and proband values of $\alpha_1 = 500, \alpha_2 = 50, \alpha_3 = 50$, or (B) the the Figure 1 exon skipping simulation with cohort values of $\alpha_1 = 1, \alpha_2 = 500, \alpha_3 = 500$ and proband values of $\alpha_1 = 50, \alpha_2 = 500, \alpha_3 = 500$.

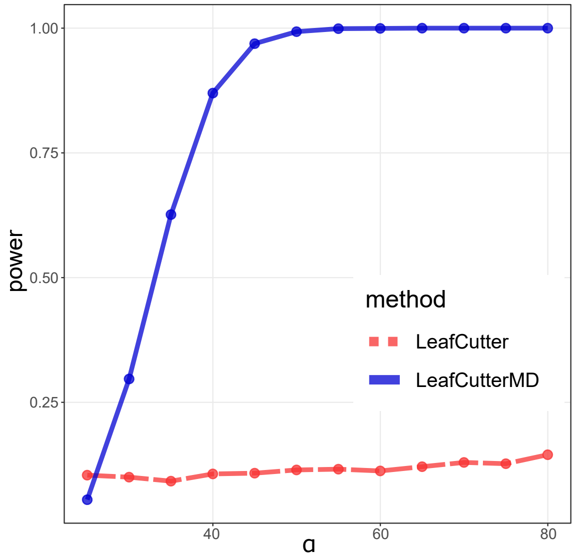


Figure S2: Power results in simulation with varying effect size $\alpha$ in the two-intron plus one noise intron setting of Figure 4 with cohort values of $\alpha_1 = 1, \alpha_2 = 500, \alpha_3 = 5000$ and proband values of $\alpha_1 = \alpha, \alpha_2 = 500, \alpha_3 = 5000$.
